# Diagnostic Challenges of Lyme Co-infections: Lessons From a Lyme and Herpes Simplex Virus-1 (HSV-1) Cocktail

**DOI:** 10.7759/cureus.60213

**Published:** 2024-05-13

**Authors:** Mannat K Bhatia, Mohamed Abdelbaky, Lokesh Lahoti

**Affiliations:** 1 Department of Internal Medicine, Rutgers Robert Wood Johnson Medical School, Saint Peter's University Hospital, New Brunswick, USA

**Keywords:** bell's palsy, diagnostic challenge, facial palsy, misdiagnosis, co-infection, hsv-1, lyme disease

## Abstract

Lyme borreliosis (LB) is a complex tick-borne illness with diverse presentations. We report a case of LB meningitis with herpes simplex virus-1 (HSV-1) co-infection in a 55-year-old woman initially presenting with isolated facial nerve palsy. This case illustrates the multifaceted diagnostic challenges associated with Lyme co-infections. It emphasizes the need for thorough testing to identify all potential pathogens and the importance of differentiating between true co-infection and incidental HSV-1 reactivation. Understanding these complexities is crucial for guiding appropriate treatment decisions.

## Introduction

Lyme disease, caused by the spirochete bacterium *Borrelia burgdorferi*, is a vector-borne illness affecting multiple organ systems [1]. It can manifest with diverse clinical presentations, including skin lesions (erythema migrans), musculoskeletal complaints, neurological complications (Lyme neuroborreliosis), and cardiac involvement (Lyme carditis) [1-3]. Diagnosis of Lyme disease can be challenging due to the lack of a gold standard test and potential cross-reactivity with other infections [4]. This can lead to misdiagnosis, with some studies reporting overdiagnosis based on serologic testing alone [5]. Antibiotic therapy remains the mainstay of treatment for Lyme disease [6,7].

This case report describes a 55-year-old woman presenting with severe headaches, facial asymmetry, neck stiffness, nausea, and photophobia. She had a recent viral infection, and examination revealed signs of Bell's palsy. Cerebrospinal fluid (CSF) serology was positive for *Borrelia burgdorferi*, followed by a positive serologic test for herpes simplex virus-1 (HSV-1). This case highlights the diagnostic challenges associated with co-infection of Lyme disease and HSV-1.

## Case presentation

A 55-year-old woman presented to our hospital with severe, persistent headaches and facial asymmetry. She also reported concurrent neck stiffness, nausea, and photophobia. Her medical history was significant for a recently resolved viral infection and migraines. She denied recent travel or tick bites.

**Figure 1:**
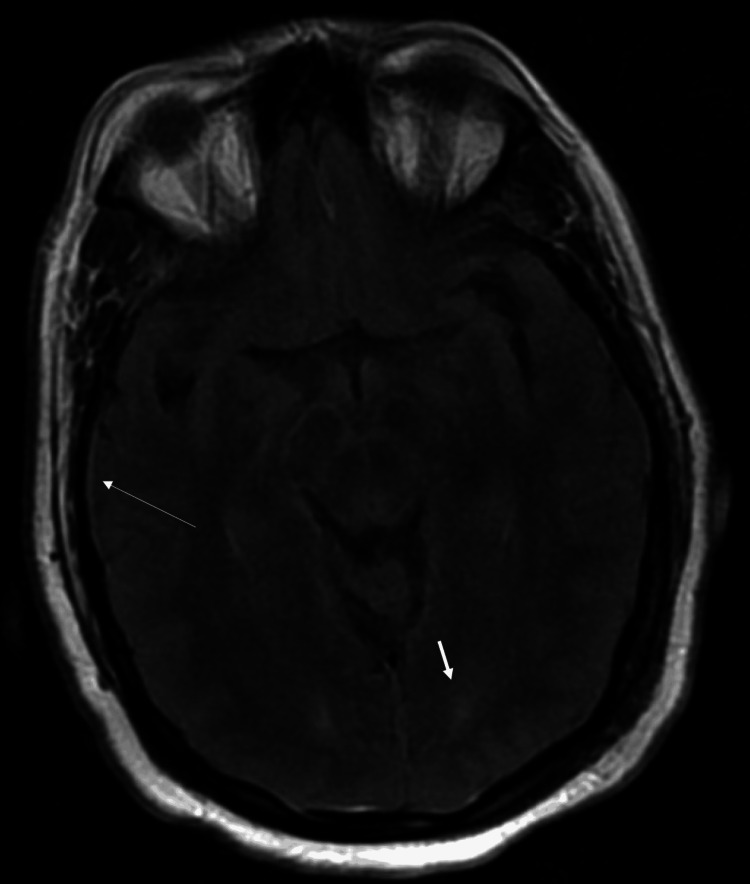
MRI of the brain w/ and w/o contrast T2-weighted MRI of the brain image with and without contrast reveals thickening of the dura mater (long arrowhead), suggesting inflammation. Additionally, nonspecific white matter hyperintensities are seen bilaterally in the parietal lobes (short arrowheads).

**Figure 2:**
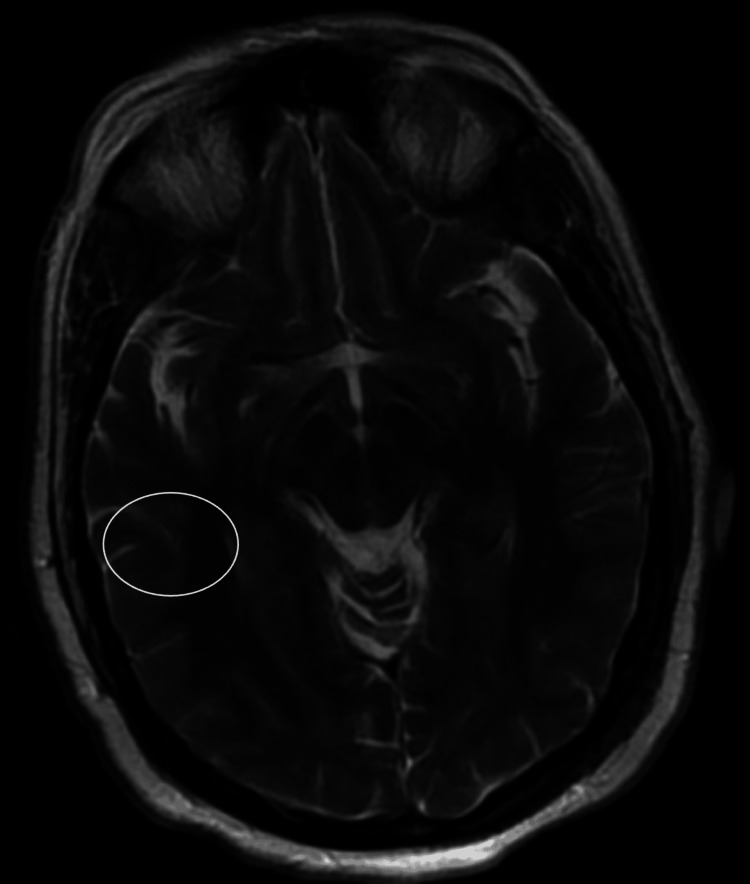
MRI of the brain w/ and w/o contrast T2-weighted MRI of the brain image highlights leptomeningeal enhancement (circled), a sign of meningeal inflammation.

Physical examination revealed a left-sided facial droop, inability to close her left eye, mild left labial fold flattening, and sparing of the left forehead. Initially, the patient was suspected of having a stroke and underwent a head CT scan, which was unremarkable. MRI revealed leptomeningeal enhancement, dural thickening, and faint linear enhancement involving the left seventh/eighth cranial nerve complex (Figure 1 and Figure 2). Additionally, nonspecific bilateral parietal skull abnormalities were noted, raising concern for possible bone metastasis.

Viral meningitis with malignancy was suspected as a differential diagnosis. A bone biopsy was performed and ruled out malignancy. Prednisone was initiated for presumed Bell's palsy, but the patient's symptoms continued to worsen.

Lumbar puncture revealed an elevated opening pressure of 43 cm H2O with moderate lymphocytic pleocytosis. Lyme disease serology in the CSF was positive. The patient's clinical improvement remained minimal, prompting a repeat lumbar puncture. The second lumbar puncture showed an opening pressure of 32 cm H2O. CSF polymerase chain reaction (PCR) for Lyme disease was also positive, while cryptococcal antigen and quantitative human immunodeficiency virus (HIV) tests were negative. Additionally, HSV-1 serology returned positive.

The patient was then treated with intravenous (IV) ceftriaxone 2 grams daily for three weeks and IV acyclovir for 14 days. She demonstrated clinical improvement, and her headaches resolved. On follow-up, she reported continued improvement.

## Discussion

The Centers for Disease Control and Prevention (CDC) estimates that approximately 476,000 people in the United States are diagnosed and treated for Lyme disease annually [8]. This rapid increase highlights the growing need to understand complex aspects of the disease, including co-infections. While the term "co-infection" often refers to illnesses transmitted alongside Lyme disease, it can also encompass opportunistic infections that arise due to Lyme-induced immune compromise. This case demonstrates the challenges associated with co-infection of Lyme disease and HSV-1, particularly regarding diagnosis and potential interactions between these pathogens.

HSV infections, often sexually transmitted, are widespread and can lead to lifelong latent infections with sporadic reactivation of viral shedding [9,10]. HSV-1, while commonly associated with oral lesions, can also cause more severe complications such as encephalitis [11].

Lyme disease diagnosis faces challenges due to the limitations of common serologic tests. These tests often rely on detecting antibodies against a single strain of *Borrelia burgdorferi* [12]. Western blot analysis can aid in diagnosing acute facial palsy by detecting early IgM antibodies associated with Lyme disease [13]. However, persistent antibody responses even after antibiotic treatment for Lyme arthritis can complicate the differentiation between active disease and other inflammatory conditions [14]. Furthermore, diverse immune responses to various *Borrelia* species antigens create additional diagnostic hurdles [15]. Notably, serologic testing for Lyme disease carries a low positive predictive value in regions with low disease prevalence, emphasizing the importance of confirmatory laboratory testing [16].

The progression of Lyme disease to the early disseminated stage with musculoskeletal, neurological, or cardiac symptoms underscores the need to consider this diagnosis in various clinical presentations, particularly in endemic regions [17]. The geographic expansion of Lyme disease across the United States further highlights the necessity for increased awareness and broader diagnostic considerations [18].

This case of a 55-year-old woman presenting with severe headaches, facial asymmetry, neck stiffness, nausea, and photophobia underscores the complexities of diagnosing co-infections. While laboratory investigations confirmed Lyme disease and HSV-1, it is crucial to acknowledge reports of false-positive Lyme serologic tests in the context of recent primary varicella-zoster virus (VZV) and HSV-2 infections [19,20]. This raises the possibility of misdiagnosis and highlights the need for a comprehensive diagnostic approach that considers the potential for cross-reactivity with other infections.

## Conclusions

This case demonstrates the importance of considering Lyme neuroborreliosis in patients with peripheral facial nerve palsy, even without a confirmed tick bite. Co-infections should be suspected in Lyme disease patients who do not improve with standard antibiotic treatment. Atypical presentations, unclear history, and co-infections, as seen in this case, increase the risk of misdiagnosis. Therefore, a comprehensive approach is crucial for the accurate diagnosis of Lyme disease and co-infections, especially when presentations are unusual or treatment response is suboptimal.
